# Gene Therapy Corrects Mitochondrial Dysfunction in Hematopoietic Progenitor Cells and Fibroblasts from *Coq9^R239X^* Mice

**DOI:** 10.1371/journal.pone.0158344

**Published:** 2016-06-24

**Authors:** Eliana Barriocanal-Casado, Cristina Cueto-Ureña, Karim Benabdellah, Alejandra Gutiérrez-Guerrero, Marién Cobo, Agustín Hidalgo-Gutiérrez, Juan José Rodríguez-Sevilla, Francisco Martín, Luis C. López

**Affiliations:** 1 Departamento de Fisiología, Facultad de Medicina, Universidad de Granada, Granada, Spain; 2 Instituto de Biotecnología, Centro de Investigación Biomédica, Universidad de Granada, Granada, Spain; 3 Genomic Medicine Department. GENYO, Centre for Genomics and Oncological Research, Pfizer-University of Granada-Andalusian Regional Government, Granada, Spain; Swedish Neuroscience Institute, UNITED STATES

## Abstract

Recent clinical trials have shown that *in vivo* and *ex vivo* gene therapy strategies can be an option for the treatment of several neurological disorders. Both strategies require efficient and safe vectors to 1) deliver the therapeutic gene directly into the CNS or 2) to genetically modify stem cells that will be used as Trojan horses for the systemic delivery of the therapeutic protein. A group of target diseases for these therapeutic strategies are mitochondrial encephalopathies due to mutations in nuclear DNA genes. In this study, we have developed a lentiviral vector (CCoq9WP) able to overexpress *Coq9* mRNA and COQ9 protein in mouse embryonic fibroblasts (MEFs) and hematopoietic progenitor cells (HPCs) from *Coq9*^*R239X*^ mice, an animal model of mitochondrial encephalopathy due to primary Coenzyme Q (CoQ) deficiency. Ectopic over-expression of *Coq9* in both cell types restored the CoQ biosynthetic pathway and mitochondrial function, improving the fitness of the transduced cells. These results show the potential of the CCoq9WP lentiviral vector as a tool for gene therapy to treat mitochondrial encephalopathies.

## Introduction

Mitochondrial diseases are a heterogeneous group of rare diseases that generally affect mitochondrial oxidative phosphorylation (OXPHOS) system directly or indirectly. These disorders can be due to mutations in mitochondrial DNA (mtDNA), which cause maternally sporadic or disorders inherited through the maternal lineage, or due to mutations in nuclear DNA (nDNA), which show a Mendelian pattern of inheritance. Because the human brain has such high-energy dependence, almost all presentations of mitochondrial disease contain neurologic symptoms. Thus, mitochondrial encephalopathy is the most common neurometabolic disorder, but current therapies are frequently inadequate, inefficient and mostly palliative [1].

Primary Coenzyme Q10 (CoQ_10_) deficiency is a mitochondrial disorder that is presented in some cases as an encephalopathic form [2], which is recapitulated in the *Coq9*^*R239X*^ mouse model. *Coq9*^*R239X*^ mice have a dysfunctional COQ9 protein, which leads to a severe reduction in COQ7, an enzyme of the CoQ biosynthetic pathway that catalyzes the hydroxylation of demethoxyubiquinone (DMQ) to produce 5-hydroxyquinone (5-HQ) (S1 Fig) [3, 4]. As a result, tissues from *Coq9*^*R239X*^ mice accumulate DMQ and have a severe reduction in CoQ levels, which cause a reduction in bioenergetics performance and increased oxidative damage in the cerebrum. As a consequence, *Coq9*^*R239X*^ mice show reactive astrogliosis and spongiform degeneration with early death [3].

Vector-mediated gene transfer is a promising strategy to treat monogenic diseases that affect the central nervous system (CNS). However, the blood brain barrier (BBB) can exclude the vast majority of gene transfer vehicles from reaching the CNS via the vasculature. For this reason, some strategies have been developed in the last decades in order to allow gene vectors to reach the CNS. These strategies include direct delivery of gene transfer vectors, such as lentivirus (LVs) [5] or adeno-associated virus (AAV) [6] directly into various compartments of the brain; or the use of *ex vivo* genetically modified hematopoietic stem cells (HSCs), which will migrate into the CNS and differentiate into microglia to produce the therapeutic effects [7]. Both strategies have reached clinical trials using LVs for the treatment of Parkinson’s Disease [8] and leukodystrophies [9, 10], respectively. The work reported by Palfi and colleagues demonstrated that direct applications of LVs into the human CNS is safe and can improve the levels of dopaminergic activity [8]. In a more robust approach, Biffy and coworkers demonstrated that gene modified HSCs (GM-HSCs) can be an optimal trojan horse to deliver therapeutic proteins through the body and specifically into the CNS [11, 12]. These authors showed that transplantation of GM-HSCs was able to normalize the neuropathological alterations of *Arsa*^*–/–*^mice and that the microglia derived from these GM-HSCs was the exclusive source of the lysosomal enzyme arylsulfatase A (ARSA) in the CNS. They also observed enzyme transfer and robust cross-correction of neural cell targets *in vivo* [11, 12]. Importantly, metachromatic leukodystrophy (MLD) patients treated with LVs-ARSA-HSCs have shown impressive clinical benefits [9, 10].

The results of Palfi and Biffi opened the possibility to use direct inoculation of LVs or transplantation of GM-HSCs for the treatment of mitochondrial encephalopathies due to mutations in nDNA genes. In this manuscript we aimed to study the feasibility of treating mitochondrial encephalopathies by gene therapy strategies using the *Coq9*^*R239X*^ mice as model for these diseases. We therefore analyzed whether COQ9 could be overexpressed in relevant target cells, mouse embryonic fibroblasts (MEFs) and HPCs, and study whether the ectopic expression of this protein could restore the mitochondrial dysfunction observed in *Coq9*-mutant cells.

## Materials and Methods

### Lentiviral vectors constructs, vector production and determination of vector copy number per cell

The CCoq9WP LV plasmid was constructed by standard cloning techniques using PstI/BamHI restriction enzymes to replace the eGFP in the CEWP backbone [13] for the Coq9 cDNA (obtained by gene synthesis from Genscript).

Vector production was performed as previously described [13]. Briefly, fast growing 293T cells were plated on petri-dishes (Sarsted,Newton, NC), 24h later, the vector (CCoq9WP), the packaging (pCMVΔR8.91) and the envelope (pMD2.G) plasmids were transfected using LipoD293 (SignaGen, Gainthersburg, MD, USA). The pCMVΔR8.91 and the pMD2.G plasmids (http://www.addgene.org/Didier_Trono) are described elsewhere [14]. Viral supernatants were collected 48h after transfection and the particles were frozen or concentrated by ultrafiltration at 2000 g and 4°C, using 100 Kd centrifugal filter devices (Amicon Ultra-15, Millipore, Billerica, MA) [15]. LVs particles supernatant were used to transduce MEF or mHPCs by adding different volumes to the cell cultures in order to achieve the desired multiplicities of infection (MOI). The medium was changed after 12 hours of incubation.

Vector titration was estimated on K562 cells by incubating 10^5^ cells with 1 μl, 10μl and 100 μl of viral supernatant. Transduced cells were lysed and DNA extracted after 7–10 days post-transduction. Vector copy number per cell (vcn/c) was determined using Q-PCR using the primer pair: WPRErev: 5’-cggaattgtcagtgcccaaca-3’ y WPREfw: 5’-ggtgtgcactgtgtttgctga-3’. Control genomic DNA of K562 cells (10^5^) were mixed with 10-fold increasing amounts of CCoq9WP plasmid DNA (10^2^ up to 10^7^ copies) to establish the standard curve in each experiment. Titre (Transducing units/ml) were estimated taking into account the calculated vcn/c, the viral volume used and the initial cell number (10^5^).

### Mouse model

The *Coq9*^*R239X*^ mouse model (http://www.informatics.jax.org/allele/key/829271) was previously generated in collaboration with Ingenious Targeting Laboratory and characterized in our lab under mix of C57BL/6N and C57BL/6J genetic background [3, 4]. *Coq9*^*R239X/+*^ mice were crossbred in order to generate *Coq9*^*+/+*^, *Coq9*^*R239X/+*^, *Coq9*^*R239X/R239X*^ (referred in the article as *Coq9*^*R239X*^). Only homozygous wild-type and mutant mice were used in the study.

Mice were housed in the Animal Facility of the University of Granada under an SPF zone with lights on at 7:00 AM and off at 7:00 PM. Mice had unlimited access to water and rodent chow. All experiments were performed according to a protocol approved by the Institutional Animal Care and Use Committee of the University of Granada (procedures 92-CEEA-OH-2015) and were in accordance with the European Convention for the Protection of Vertebrate Animals used for Experimental and Other Scientific Purposes (CETS # 123) and the Spanish law (R.D. 53/2013).

### MEFs isolation and transduction from donor mice

MEFs from *Coq9*^*+/+*^ and *Coq9*^*R239X*^ mice were grown in complete medium (high glucose DMEM-GlutaMAX medium supplemented with 10% FBS, 1% MEM non-essential amino acids and 1% antibiotics/antimycotic). For transduction, 0.5–1 x 10^5^ MEFs were exposed to increasing doses of the LVs supernatant in DMEM medium in the absence of serum during 12 hours. After that, MEFs were cultured in complete medium during 7 to 20 days.

### Bone marrow isolation and transduction from donor mice

The bone marrow was isolated from 4–6 weeks old donor mice (*Coq9*^*+/+*^ or *Coq9*^*R239X*^). Bone marrow cells were harvested from euthanized donor mice by CO_2_ asphyxiation followed by cervical dislocation. The cells were flushed out of the 2 femurs and 2 tibias under sterile conditions, passed through a 40 μm cell strainer and treated with ammonium chloride solution (Stem Cell Technologies) to eliminate the red blood cells. Cells were stained with anti-mouse Ly-6A/E (Sca-1)-PE (eBioscience, San Diego, CA), washed with AutoMACS buffer and incubated with Anti-PE MicroBeads (Miltenyi Biotec) following the manufacturer´s instructions. An enriched mouse hematopoietic progenitor cells (HPCs) fraction was obtained after a magnetic separation using AutoMACS Pro Separator (Miltenyi Biotec). For transduction, 1x10^6^ mHPCs were incubated with LVs supernatant at MOI = 200 in Stem-Span Serum-Free Expansion Medium (StemCell Technologies) supplemented with 1% FBS, 1% penicillin/streptomycin and cytokines (mouse IL-3, murine SCF, human FMS-like tyrosine kinase 3 ligand [hFlt3L] and human IL-6) for 12 hours. After that, mHPCs were cultured in complete Stem-Span medium during 7 to 14 days.

### Quantification of CoQ_9_ and DMQ_9_ levels in MEFs and mHPCs

After lipid extraction from homogenized cultured MEFs and mHPCs, CoQ_9_ and DMQ_9_ levels were determined via reversed-phase HPLC coupled to electrochemical (EC) detection [16]. The results were expressed in ng CoQ/mg prot and as DMQ_9_/CoQ_9_ ratio.

### Gene expression analyses

Total cellular RNA from frozen cell pellets were extracted and electrophoresed in agarose 1.5% to check RNA integrity. RNA from MEFs and mHPCs was extracted with Real Total RNA Spin Plus Kit (Real). Total RNA was quantified by optical density at 260/280 nm and was used to generate cDNA with High Capacity cDNA Reverse Transcription Kit (Applied Biosystems). Amplification was performed with quantitative real-time PCR, by standard curve method, with specific Taqman probes (from Applied Biosystems) for the targeted gene mouse Coq9 (Mm00804236_m1) and the mouse Hprt probe as a standard loading control (Mm01545399_m1).

### Sample preparation and Western blot analysis in cells

Cells were collected, washed twice with 1× PBS and homogenated in RIPA buffer freshly supplemented with proteases inhibitors. To detect COQ7 protein, 60 μg of proteins from the sample extracts was electrophoresed in 12% Mini-PROTEAN TGX^™^ precast gels (Bio-Rad) using the electrophoresis system mini-PROTEAN Tetra Cell (Bio-Rad). To detect COQ9 protein, 60 μg of proteins from sample extracts was prepared in XT sample buffer + XT-reducing agent (Bio-Rad) and electrophoresed in a 10% Criterion^™^ XT precast gel (Bio-Rad) using MOPS running buffer and the electrophoresis system Criterion Cell (Bio-Rad). In all experiments, proteins were transferred onto PVDF 0.45 μm membranes using a mini Trans-blot Cell (Bio-rad) or Trans-blot Cell (Bio-Rad) and probed with target antibodies. Protein-antibody interactions were detected with peroxidase-conjugated horse anti-mouse, anti-rabbit or anti-goat IgG antibodies using Amersham ECL^™^ Prime Western Blotting Detection Reagent (GE Healthcare, Buckinghamshire, UK). Band quantification was carried out using an Image Station 2000R (Kodak, Spain) and a Kodak 1D 3.6 software. COQ7 and COQ9 protein band intensity was normalized to VDAC1, and the data expressed in terms of percent relative to wild-type mice [4].

### Flow cytometry

mHPCs were collected, washed with cold PBS containing 2% FBS and stained with fluorochrome conjugated monoclonal antibodies anti-mouse CD11b-APC (BD-Bioscience) or anti-mouse Ly-6A/E (Sca-1)-PE (eBioscience, San Diego, CA). Cells were acquired and analyzed on a FACS Canto II flow cytometer (Becton Dickinson, Franklin Lakes, NJ) using the FACS Diva software (BD Biosciences, Bedford, MA).

### Assessment of mitochondrial function

Oxygen consumption rate (OCR) was measured in adherent fibroblasts with a XF24 Extracellular Flux Analyzer (Seahorse Bioscience, Billerica, MA, USA). Each cell line was seeded in 6 wells of a XF 24-well cell culture microplate (Seahorse Bioscience) at a density of 5×10^4^ cells/well in 250 μL of DMEM and incubated for 24 h at 37°C in 5% CO_2_ atmosphere. After replacing the growth medium with 525 μL of bicarbonate-free DMEM pre-warmed at 37°C cells were preincubated for 1 hour in a CO_2_ free incubator before starting the assay procedure [17].

After baseline measurements, OCR was measured after sequentially adding to each well 75 μL of oligomycin, 75 μl of carbonyl cyanide 4-(trifluoromethoxy) phenylhy-drazone (FCCP) and 75 μl of rotenone and antimycin, to reach working concentrations of 1 μM, 0,75 μM, and 1 μM, respectively [17].

Spare respiratory capacity was calculated according to the User Manual XF cell mito stress test kit (SeaHorse Biosciences). Spare respiratory capacity = (Maximal respiration)—(Basal Respiration). Non-mitochondrial respiration was subtracted in both maximal respiration and basal respiration. Spare respiratory capacity provides an idea of the cells maximum ATP production; therefore cells with a higher capacity have a greater ability to respond to stress.

### Real time analysis of cells adhesion and growth

Adhesion and proliferation of *Coq9*^*+/+*^ *-*MEFs, *Coq9*^*R239X*^ -MEFs and transduced *Coq9*^*R239X*^ -MEFs were measured using the xCelligence real-time cell analyzer system from Roche (Roche Applied Science, Penzberg, Germany, www.roche.com). 1000 cells/well were added to 16-well E-plates and analyzed as described previously [18]. Briefly, the E-plates loaded with the different cells were placed on the device station in the incubator (5% CO_2_ at 37°C) for continuous recording of impedance (a direct measure of the amount of cells adhered to the E-plate at each time point), as reflected by cell index.

### Statistical analysis

All statistical analyses were performed using the Prism 6 scientific software. Data are expressed as the mean ± SD of four-six experiments per group. A one-way ANOVA with a Tukey *post hoc* test was used to compare the differences between three experimental groups. A *P*-value of 0.05 was considered to be statistically significant.

## Results

### Overexpression of COQ9 in MEFs and mHPCs from *Coq9*^*R239X*^ mice

**Fig 1:**
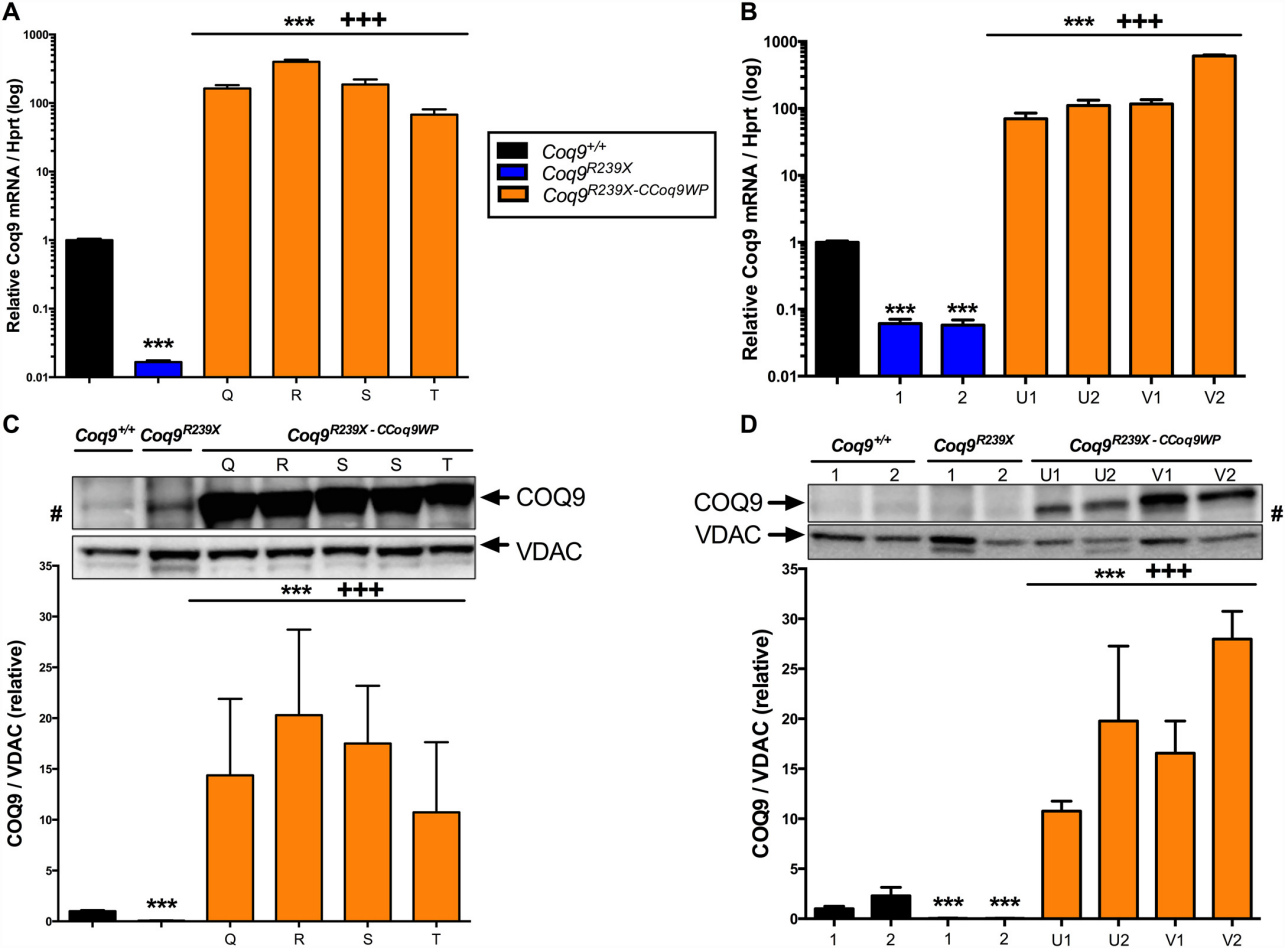
Transduction with CCoq9WP vector increases the levels of Coq9 mRNA and COQ9 protein in MEFs and mHPCs from *Coq9*^*R239X*^ mice. Coq9 mRNA levels in MEFs (*A*) and mHPCs (*B*). COQ9 protein levels in MEFs (*C*) and mHPCs (*D*). Q: 1 hit in MEFs, 200 μl, non-concentrated; R: 1 hit in MEFs, 100 μl, 10x-concentrated; S: 1 hit in MEFs, 25 μl, 10x-concentrated; T: 1 hit in MEFs, 5 μl, 10x-concentrated; U: 1 hit in HSCs, 400 μl, 23.5x-concentrated; V: 2 hits in HSCs 400 μl, 23.5x-concentrated; 1: 7–9 days after transduction in HSCs; 2: 12–16 days after transduction in HSCs. Data are expressed as mean ± SD. ***P < 0.001, *Coq9*^*R239X*^ and *Coq9*^*R239X-CCoq9WP*^ cells versus *Coq9*^*+/+*^ cells; +++*P* < 0.001, *Coq9*^*R239X-CCoq9WP*^ cells versus *Coq9*^*R239X*^ cells; (one-way ANOVA with a Tukey's post hoc test; n = 4–6 for each group). #: a non-specific band is detected in *Coq9*^*R239X*^ cells with the anti-COQ9 antibody.

We generated a LV expressing *Coq9* through the CMV promoter by insertion of the *Coq9* cDNA into the CEWP vector backbone [13] (see M&M for details). MEFs and mHPCs derived from *Coq9*^*R239X*^ mice were transduced with CCoq9WP LVs particles at increasing MOI and investigated whether transduced cells produce supraphysiological levels of *Coq9* mRNA and COQ9 protein (Fig 1). In both cell models, the transduction with CCoq9WP produced levels of *Coq9* mRNA 100–700 times higher than the levels observed in control *Coq9*^*+/+*^ cells (Fig 1A and 1B). As a result, the levels of COQ9 protein were 10–30 times higher in transduced *Coq9*^*R239X*^ cells than in control *Coq9*^*+/+*^ cells (Fig 1C and 1D). Therefore, the CCoq9WP LV was able to increase the *Coq9* mRNA and COQ9 protein beyond control values in both MEFs and mHPCs from *Coq9*^*R239X*^ mice.

### Overexpression of COQ9 restores the normal function of the CoQ biosynthetic pathway in MEFs and mHPCs from *Coq9*^*R239X*^ mice

**Fig 2:**
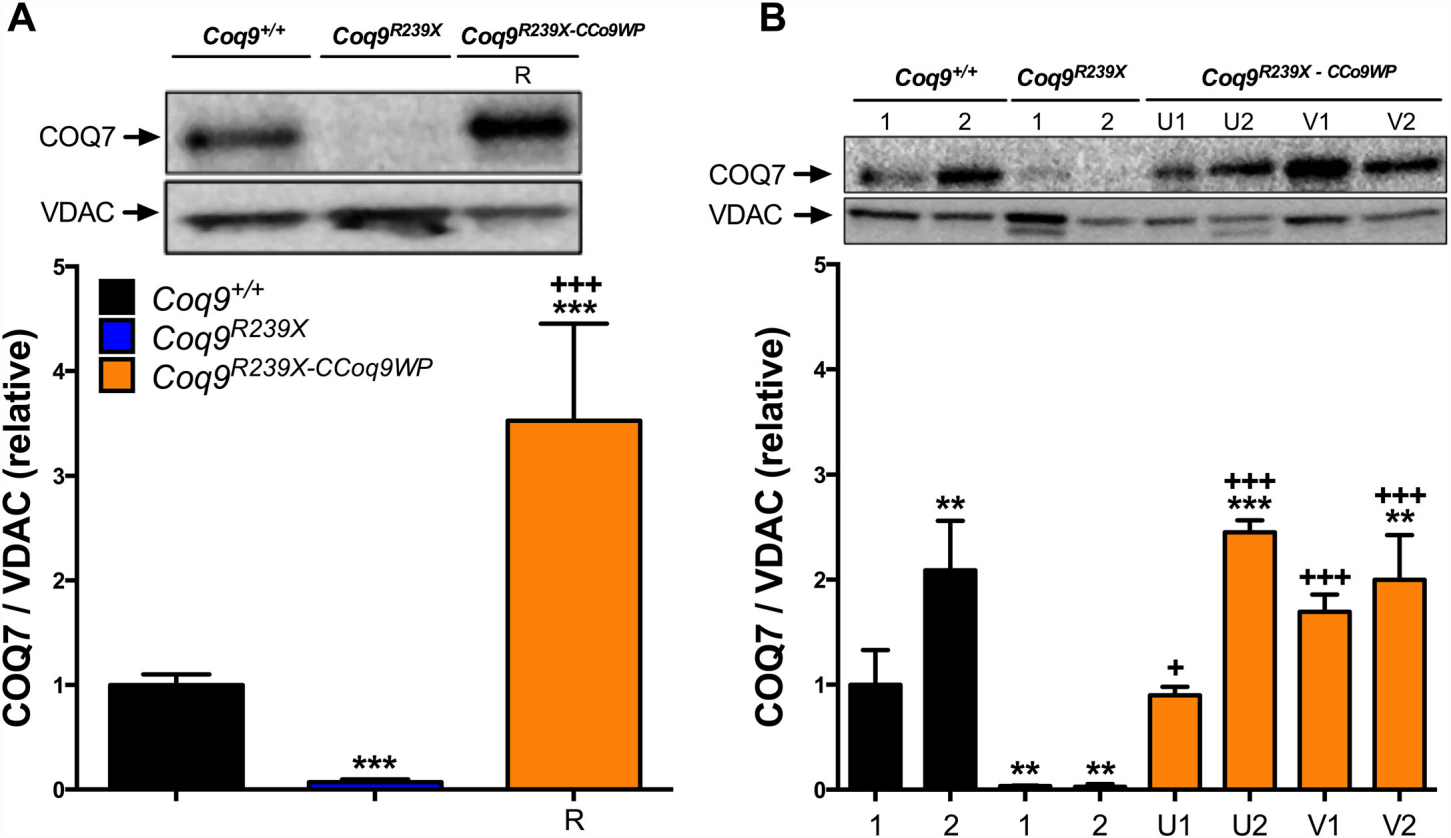
Overexpression of COQ9 in *Coq9*^*R239X*^ MEFs and mHPCs increases the levels of COQ7. Levels of COQ7 in MEFs (*A*) and mHSCs (*B*). R: 1 hit in MEFs, 100 μl, 10x-concentrated; U: 1 hit in HPCs, 400 μl, 23.5x-concentrated; V: 2 hits in HPCs 400 μl, 23.5x-concentrated; 1: 7–9 days after transduction in HSCs; 2: 12–16 days after transduction in HSCs. Data are expressed as mean ± SD. **P < 0.01, *Coq9*^*R239X*^ and *Coq9*^*R239X-CCoq9WP*^ cells versus *Coq9*^*+/+*^ cells; ***P < 0.001, *Coq9*^*R239X*^ and *Coq9*^*R239X-CCoq9WP*^ cells versus *Coq9*^*+/+*^ cells; +*P* < 0.05, *Coq9*^*R239X-CCoq9WP*^ cells versus *Coq9*^*R239X*^ cells; +++*P* < 0.001, *Coq9*^*R239X-CCoq9WP*^ cells versus *Coq9*^*R239X*^ cells; (one-way ANOVA with a Tukey's post hoc test; n = 4–6 for each group).

**Fig 3:**
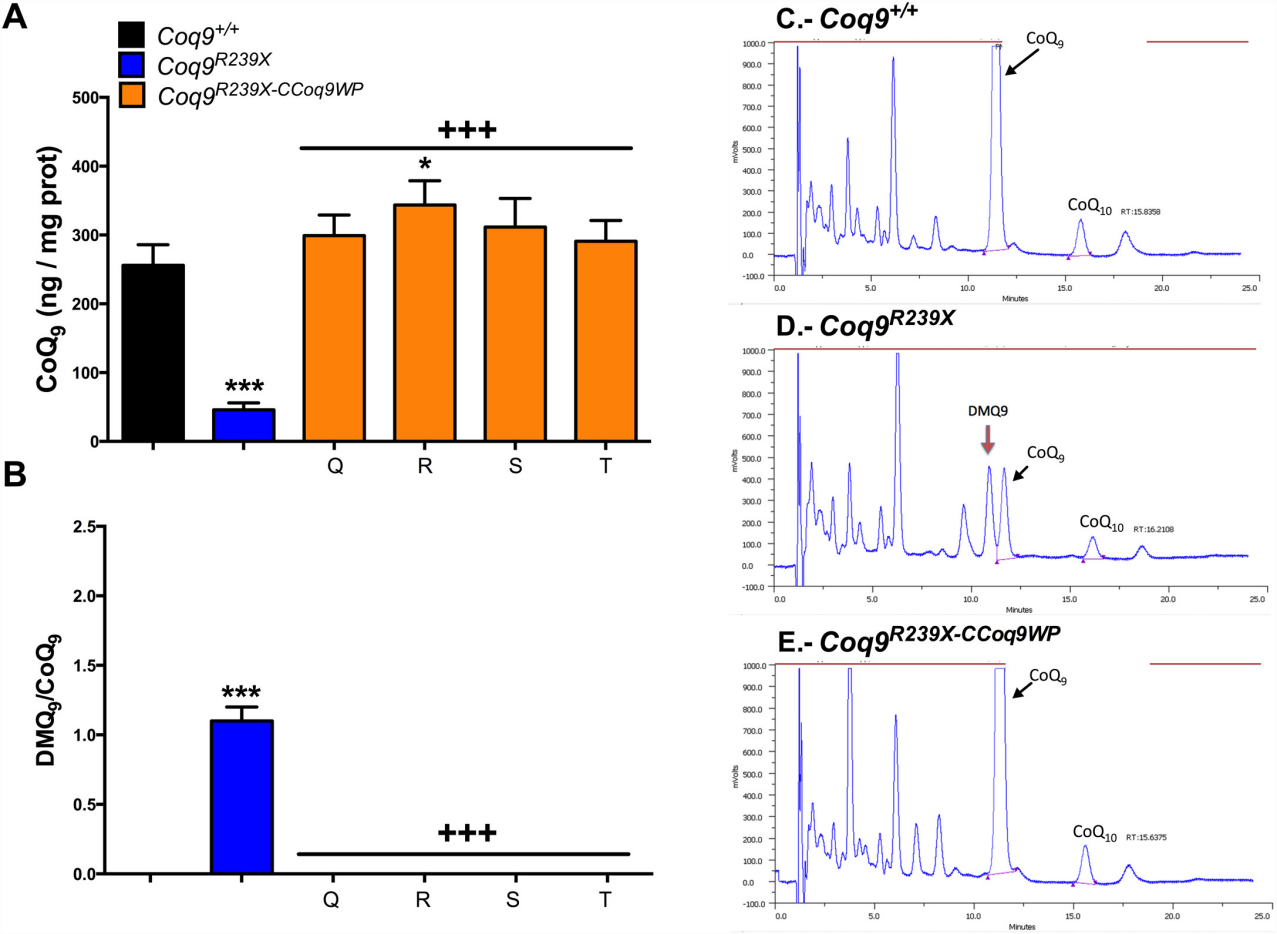
Lack of DMQ_9_ and increase of CoQ_9_ in *Coq9*^*R239X*^ MEFs after transduction with CCoq9WP vector. Levels of CoQ_9_ (*A*) and DMQ_9_/CoQ_9_ ratio (*B*). Representative chromatographs of the three different groups (*C-E*). Q: 1 hit in MEFs, 200 μl, non-concentrated; R: 1 hit in MEFs, 100 μl, 10x-concentrated; S: 1 hit in MEFs, 25 μl, 10x-concentrated; T: 1 hit in MEFs, 5 μl, 10x-concentrated. Data are expressed as mean ± SD. *P < 0.05, *Coq9*^*R239X*^ and *Coq9*^*R239X-CCoq9WP*^ cells versus *Coq9*^*+/+*^ cells; ***P < 0.001, *Coq9*^*R239X*^ and *Coq9*^*R239X-CCoq9WP*^ cells versus *Coq9*^*+/+*^ cells; +++*P* < 0.001, *Coq9*^*R239X-CCoq9WP*^ cells versus *Coq9*^*R239X*^ cells; (one-way ANOVA with a Tukey's post hoc test; n = 4–6 for each group).

**Fig 4:**
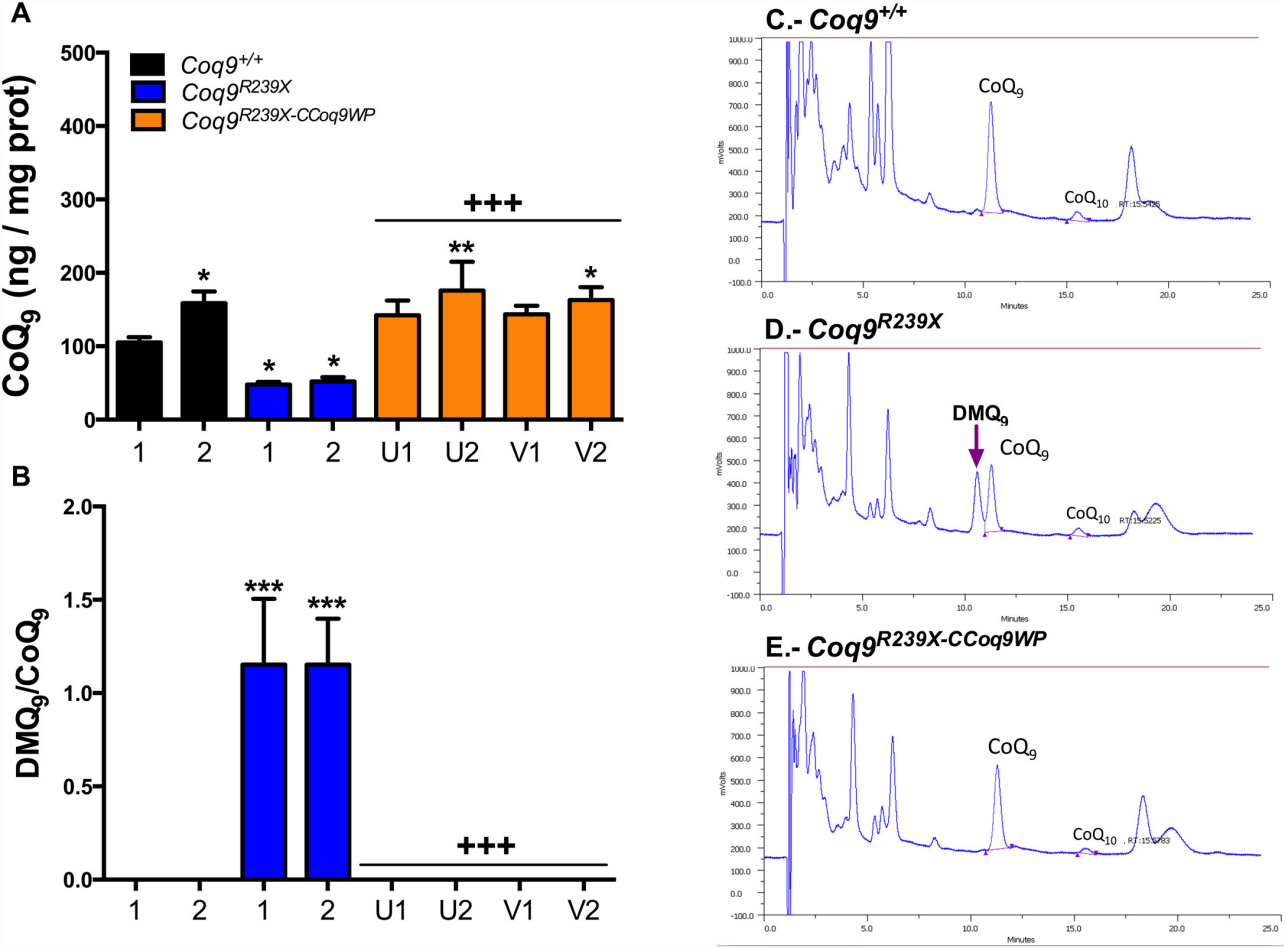
Lack of DMQ_9_ and increase of CoQ_9_ in *Coq9*^*R239X*^ mHPCs after transduction with CCoq9WP vector. Levels of CoQ_9_ (*A*) and DMQ_9_/CoQ_9_ ratio (*B*). Representative chromatographs of the three different groups (*C-E*). U: 1 hit in HSCs, 400 μl, 23.5x-concentrated; V: 2 hits in HPCs 400 μl, 23.5x-concentrated; 1: 7–9 days after transduction in HPCs; 2: 12–16 days after transduction in HSCs. Data are expressed as mean ± SD. *P < 0.05, *Coq9*^*R239X*^ and *Coq9*^*R239X-CCoq9WP*^ cells versus *Coq9*^*+/+*^ cells; **P < 0.01, *Coq9*^*R239X*^ and *Coq9*^*R239X-CCoq9WP*^ cells versus *Coq9*^*+/+*^ cells; ***P < 0.001, *Coq9*^*R239X*^ and *Coq9*^*R239X-CCoq9WP*^ cells versus *Coq9*^*+/+*^ cells; +++*P* < 0.001, *Coq9*^*R239X-CCoq9WP*^ cells versus *Coq9*^*R239X*^ cells; (one-way ANOVA with a Tukey's post hoc test; n = 4–6 for each group).

COQ9 is needed for the stability and functional activity of the hydroxylase COQ7 [3, 4]. Thus, the *Coq9*^*R239X*^ mouse model shows a severe reduction in the tissue levels of COQ7 and accumulation of DMQ_9_, the substrate of the reaction catalyzed by COQ7 (S1 Fig) [3, 4]. This pattern was also observed in MEFs and mHPCs from *Coq9*^*R239X*^ mice (Fig 2A and 2B). The overexpression of COQ9 in transduced *Coq9*^*R239X*^ MEFs and mHPCs induced an increase in COQ7 levels, which were even higher than the levels observed in control cells (Fig 2A and 2B). As a result, DMQ_9_ does not accumulate in CCoq9WP LV-transduced *Coq9*^*R239X*^ cells (Figs 3B, 3C, 3D, 3E, 4B, 4C, 4D and 4E) and the levels of CoQ_9_, the final product of the pathway, were normalized (Figs 3A and 4A). In both wild-type and CCoq9WP LV-transduced *Coq9*^*R239X*^ mHPCs, we also observe higher levels of COQ7 and CoQ_9_ over the time (Figs 2B and 4A; S1 and S2 Tables). This was in parallel to the differentiation of mHPCs during culture time (S2 Fig).

### Overexpression of COQ9 normalizes mitochondrial function and enhances fitness in MEFs from *Coq9*^*R239X*^ mice

**Fig 5:**
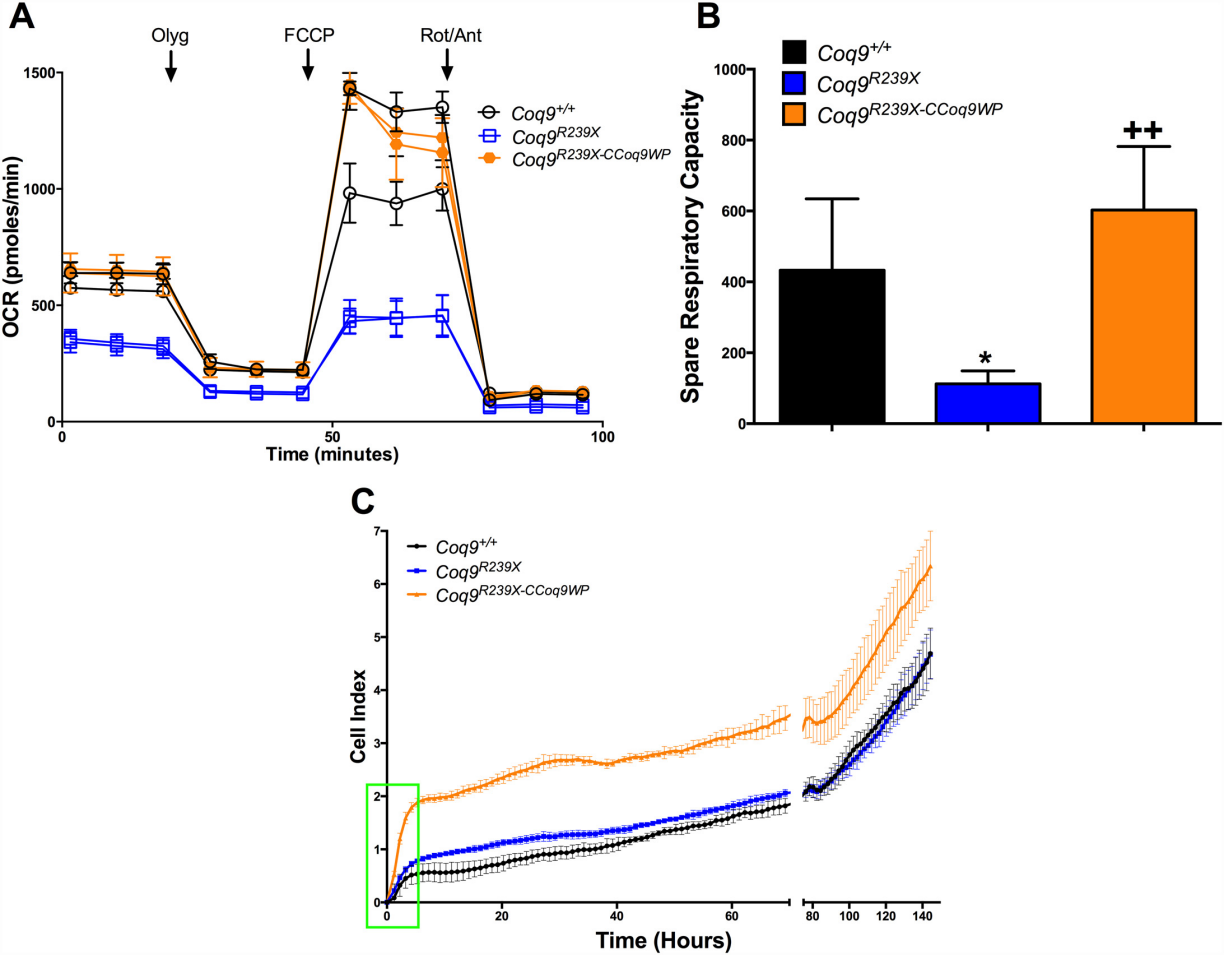
Transduction with CCoq9WP vector restores mitochondrial respiration and enhances fitness in MEFs from *Coq9*^*R239X*^ mice. Oxygen consumption rate (OCR) profile (A), spare respiratory capacity (B) and cell index (C) in MEFs. Vertical arrows indicate the time of addition of oligomycin (olyg), FCCP and rotenone/antymicin (Rot/Ant). 5×10^4^ cells were plated in each well. Data are expressed as mean ± SD. *P < 0.05, *Coq9*^*R239X*^ and *Coq9*^*R239X-CCoq9WP*^ cells versus *Coq9*^*+/+*^ cells; ++*P* < 0.01, *Coq9*^*R239X-CCoq9WP*^ cells versus *Coq9*^*R239X*^ cells; (one-way ANOVA with a Tukey's post hoc test; n = 4–6 for each group).

Skin fibroblasts of patients with primary CoQ_10_ deficiency show reduced activities of CoQ-dependent mitochondrial complexes and decreased levels of ATP [16, 19–23]. Similarly, isolated mitochondria from tissues of *Coq9*^*R239X*^ show a reduction in mitochondrial respiration [3, 4]. Therefore, we next investigated whether the increase of CoQ_9_ induces functional changes in mitochondrial bioenergetics. We assessed the mitochondrial respiration in MEFs by SeaHorse Analyzer. *Coq9*^*R239X*^ MEFs show a decrease in the global oxygen consumption rate and in the spare respiratory capacity (Fig 5A and 5B). Both alterations were normalized after transduction of *Coq9*^*R239X*^ MEFs with the CCoq9WP LV (Fig 5A and 5B). As a consequence, cell growth was increased in CCoq9WP-transduced *Coq9*^*R239X*^ compared to untransduced *Coq9*^*R239X*^ MEFs and also to wild-type MEFs (Fig 5C). The improved cell growth was restricted to the first hours after plating, indicating that over-expression of COQ9 could improve adhesion of MEFs to plastic and this could favor initial growth (see green square in Fig 5C). After that moment, the growth rate was similar in the three experimental groups (Fig 5C).

## Discussion

Mitochondrial encephalopathies are commonly caused by mutations in nDNA genes. Most of these diseases are untreatable, other than by relieving certain symptoms. GT strategies aim for restoring the function by direct transfer of genes encoding the wild-type protein into the CNS [5, 8] or by using HSCs as Trojan horses to deliver the protein or the final product into the target tissue [7]. Both strategies require efficient and safe vectors able to deliver the therapeutic gene to the CNS or to the HSCs. LVs have been used successfully for both strategies in preclinical models and clinical studies [10]. Here, we have developed a LV (CCoq9WP) able to overexpress *Coq9* mRNA and COQ9 protein in MEFs and mHPCs from *Coq9*^*R239X*^ mice, an animal model of mitochondrial encephalopathy. Importantly, the CoQ biosynthetic pathway and mitochondrial function were normalized after LV treatment.

In this study, we use MEFs and mHPCs from *Coq9*^*R239X*^ mice. This mouse model shows spongiform degeneration and severe reactive astrogliosis in the pons and diencephalon, resulting in early death [3, 4]. The cause of these pathological changes is a mitochondrial bioenergetics impairment in the cerebrum due to a severe CoQ deficiency, which is caused by a mutation in *Coq9*, also reported in patients with primary CoQ_10_ deficiency [23–25]. Overexpression of Coq9 in MEFs and mHPCs was not only well tolerated but also increased the fitness of *Coq9*^*R239X*^ MEFs, probably due to their improvement in mitochondrial function and pyrimidine biosynthesis, as well as a reduction in oxidative stress and apoptosis [16, 19, 20, 22, 26, 27]. Levels of COQ9 above of control levels were also achieved in skin fibroblasts of a patient with a mutation in *COQ9* treated with a lentivirus harboring *Coq9* cDNA [23]. This gene is essential for CoQ biosynthesis by encoding a protein, COQ9, which has been proposed to be required for the stability of COQ7, a catalytic enzyme in the CoQ biosynthetic pathway [3, 4, 28, 29]. The results presented in this manuscript further confirm the interaction COQ9-COQ7 since the overexpression of COQ9 in *Coq9*^*R239X*^ cells induced an increase in the levels of COQ7. Similar results were also obtained in human skin fibroblasts of a patient with COQ9 mutation [23]. The direct consequence of the increase of COQ7 in CCoq9WP-transduced *Coq9*^*R239X*^ cells is the complete reduction of DMQ_9_, the substrate of the reaction catalyzed by this enzyme. However, even if the levels of COQ9 and COQ7 were higher in transduced cells than in control cells (10 to 30 and 1 to 5 fold-increase, respectively), the levels of CoQ_9_, the final product of the pathway, only increased 1.5 fold compared to wild-type levels. Similar results were obtained in yeasts studies, where the overexpression of *COQ8* (*ADCK3*) induced a 2.4 fold increase of CoQ levels compared to wild-type levels [30, 31]. These results reflect the complex regulation of the CoQ biosynthetic pathway, which involves, at least, 12 different proteins. Moreover, these proteins are organized in a multiprotein complex, which allow channeling of labile/reactive intermediates, enhance catalytic efficiency, and provide a mechanism for coordinative regulation of components [4, 32, 33].

An important result for future applications is the ability of the CCoq9WP LVs to over-express up to 30 times the levels of COQ9 protein in *Coq9*^*R239X*^ MEFs and mHPCs compared to wild-type MEFs and mHPCs. These data open the possibility of using these LVs as a tool for *in vivo* and *ex vivo* GT strategies for the treatment of CoQ deficiencies. Direct inoculation of LVs (*in vivo* GT) has been proved to be efficient and safe for the treatment of several diseases in animal models [34–36] and clinical studies [8]. As an alternative strategy, generation of autologous HSCs transduced with CCoq9WP LVs (GM-HSCs) could be used as Trojan horses to deliver COQ9 protein and/or CoQ lipid to different cell types distributed through the body, including neurons. GM-HSC strategy has been proven to be efficient and safe not only in animal models of leukodystrophies [11, 12] and mitochondrial neurogastrointestinal encephalomyopathy (MNGIE) [37] but also in human clinical trials of both disorders [9, 10, 38].

In mHPCs, the levels of *Coq9* mRNA, COQ9 and COQ7 proteins and CoQ_9_ lipid were higher in the second day of cell collection (12–16 days in culture) than those levels in the first day of cell collection (7–9 days in culture). These differences may be caused by an increase on mitochondrial mass, mitochondrial function and reactive oxygen species production during cell differentiation [39–43], having, in that case, positive implication for the use of these cells in the *in vivo* transplantation.

Future experiments using *in vivo* and *ex vivo* GT strategies with these LVs will provide evidences of the best strategy for the treatment of these disorders. Protein transfer of mitochondrial proteins could be a problem for GM-HSCs but, at the same time, lipid transfer (CoQ_9_) could be more efficient and achieve cross-correction of the CoQ-deficient cells. This is important for the success of the in vivo therapy because our results in other mouse model of CoQ deficiency (*Coq9*^*Q95X*^) suggest that reaching 50% of CoQ levels in cerebral cells would be enough to avoid the encephalopathic phenotype [4]. Thus, it will be desirable to compare (or combine) GM-HSCs with *in vivo* delivery of lentiviral vectors expressing the therapeutic protein as a possible treatment for mitochondrial encephalopathies.

In summary, the experimental approach used in this study show the correction of the bioenergetics impartment in cells with primary CoQ deficiency using a LV. These results support the evaluation of the CCoq9WP LV in a preclinical study *in vivo*.

## Supporting Information

S1 Fig CoQ biosynthetic pathway. Purple box indicates the dysfunctional protein in the mouse model *Coq9*^*R239X*^. COQ9 is needed for the reaction catalyzed by COQ7. Therefore, *Coq9*^*R239X*^ mice show a severe reduction of COQ7 and accumulation DMQ, the substrate of the reaction catalyzed by COQ7.
(PDF)

S2 Fig Characteristics of the growth of mHPCs cultured for 14 days. Percentage of Sca-1 positive cells (A) and CD11b positive cells (B). Sca-1 is used as a marker of mHPCs and CD11b as a marker of macrophages. *P < 0.05 versus *Coq9*^*+/+*^ cells at day 0; **P < 0.01 versus *Coq9*^*+/+*^ cells at day 0; (one-way ANOVA with a Tukey's post hoc test; n = 4 for each group and time point).
(TIFF)

S1 Table Time effect over the levels of the analyzed biomolecules in transduced mHPCs. U: 1 hit in HPCs, 400 μl, 23.5x-concentrated; 1: 7–9 days after transduction in HPCs; 2: 12–16 days after transduction in HPCs. # P < 0.05, versus U1; ### P < 0.05, versus U1; (Student's *t* Test; n = 4–6 for each group).
(DOCX)

S2 Table Time effect over the levels of the analyzed biomolecules in transduced mHPCs. V: 2 hits in HPCs 400 μl, 23.5x-concentrated; 1: 7–9 days after transduction in HPCs; 2: 12–16 days after transduction in HPCs. # P < 0.05, versus V1; ### P < 0.05, versus V1; (Student's *t* Test; n = 4–6 for each group).
(DOCX)
