# Supplementary figures and images for: Gene Therapy Corrects Mitochondrial Dysfunction in Hematopoietic Progenitor Cells and Fibroblasts from Coq9R239X Mice
Source: PLoS One. 2016 Jun 24;11(6):e0158344. doi: 10.1371/journal.pone.0158344 (PMC4920430; doi:10.1371/journal.pone.0158344)

**Figure S1**

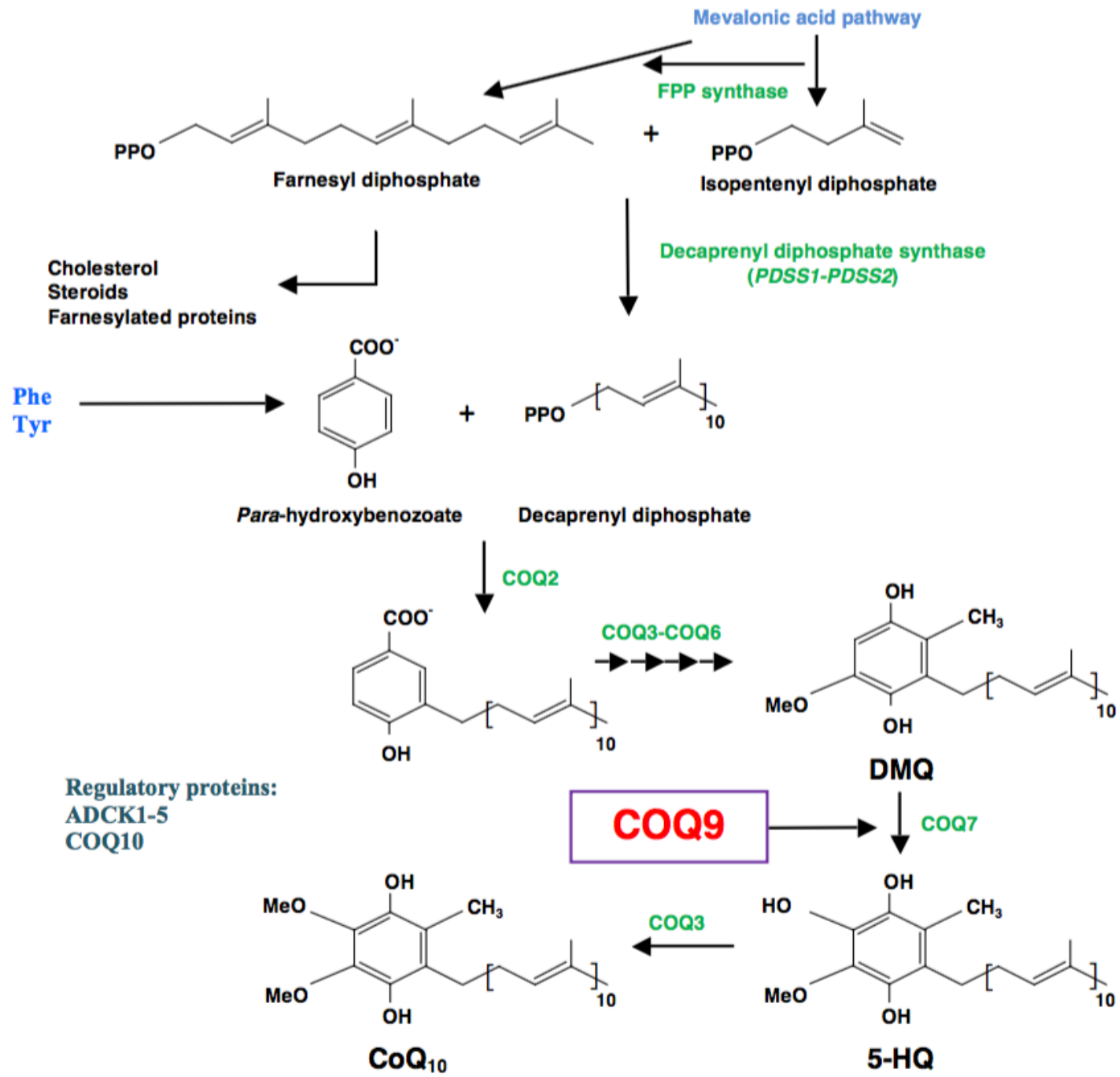

Supplement: S1 Fig — Purple box indicates the dysfunctional protein in the mouse model Coq9R239X. COQ9 is needed for the reaction catalyzed by COQ7. Therefore, Coq9R239X mice show a severe reduction of COQ7 and accumulation DMQ, the substrate of the reaction catalyzed by COQ7. (PDF) [file pone.0158344.s001.pdf]

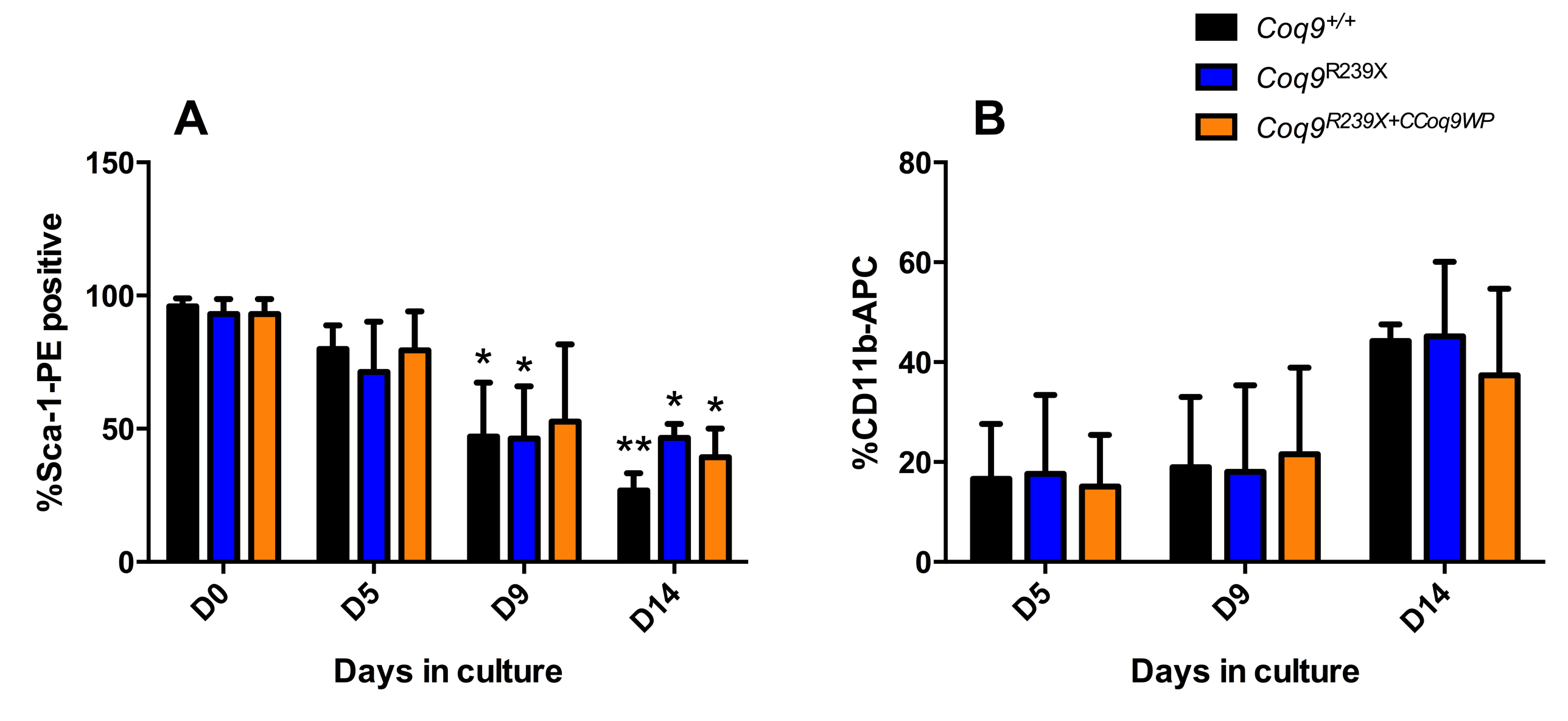

Supplement: S2 Fig — Percentage of Sca-1 positive cells (A) and CD11b positive cells (B). Sca-1 is used as a marker of mHPCs and CD11b as a marker of macrophages. *P < 0.05 versus Coq9+/+ cells at day 0; **P < 0.01 versus Coq9+/+ cells at day 0; (one-way ANOVA with a Tukey's post hoc test; n = 4 for each group and time point). (TIFF) [file pone.0158344.s002.tiff]
