# Supplementary material for: Gene Therapy Corrects Mitochondrial Dysfunction in Hematopoietic Progenitor Cells and Fibroblasts from Coq9R239X Mice
Source: PLoS One. 2016 Jun 24;11(6):e0158344. doi: 10.1371/journal.pone.0158344 (PMC4920430; doi:10.1371/journal.pone.0158344)
Supplement: S1 Table — U: 1 hit in HPCs, 400 μl, 23.5x-concentrated; 1: 7–9 days after transduction in HPCs; 2: 12–16 days after transduction in HPCs. # P < 0.05, versus U1; ### P < 0.05, versus U1; (Student's t Test; n = 4–6 for each group). (DOCX) [file pone.0158344.s003.docx]

**S1 Table. Time effect over the levels of the analyzed biomolecules in transduced mHSCs.** U: 1 hit in HSCs, 400 μl, 23.5x-concetrated; 1: 7-9 days after transduction in HSCs; 2: 12-16 days after transduction in HSCs. # P < 0.05, versus U1; ### P < 0.05, versus U1; (Student's *t* Test; n = 4-6 for each group).

|  | **U1** | **U2** |
| --- | --- | --- |
| ***Coq9* mRNA** | 70 ± 15 | 111 ± 22 ^#^ |
| **COQ9** | 11 ± 1 | 20 ± 7.5 |
| **COQ7** | 0.9 ± 0.08 | 2.5 ± 0.12 ^###^ |
| **CoQ_9_** | 142 ± 20 | 176 ± 39 |
