# Supplementary material for: Gene Therapy Corrects Mitochondrial Dysfunction in Hematopoietic Progenitor Cells and Fibroblasts from Coq9R239X Mice
Source: PLoS One. 2016 Jun 24;11(6):e0158344. doi: 10.1371/journal.pone.0158344 (PMC4920430; doi:10.1371/journal.pone.0158344)
Supplement: S2 Table — V: 2 hits in HPCs 400 μl, 23.5x-concentrated; 1: 7–9 days after transduction in HPCs; 2: 12–16 days after transduction in HPCs. # P < 0.05, versus V1; ### P < 0.05, versus V1; (Student's t Test; n = 4–6 for each group). (DOCX) [file pone.0158344.s004.docx]

**S2 Table. Time effect over the levels of the analyzed biomolecules in transduced mHSCs.** V: 2 hits in HSCs 400 μl, 23.5x-concetrated; 1: 7-9 days after transduction in HSCs; 2: 12-16 days after transduction in HSCs. # P < 0.05, versus V1; ### P < 0.05, versus V1; (Student's *t* Test; n = 4-6 for each group).

|  | **V1** | **V2** |
| --- | --- | --- |
| ***Coq9* mRNA** | 117 ± 19 | 609 ± 23 ^###^ |
| **COQ9** | 17 ± 3.2 | 28 ± 2.8 ^#^ |
| **COQ7** | 1.7 ± 0.17 | 2 ± 0.43 |
| **CoQ_9_** | 143 ± 12 | 163 ± 18 |
